# DDBJ update in 2025: system integration for global data-sharing including pathogen surveillance

**DOI:** 10.1093/nar/gkaf1273

**Published:** 2025-11-24

**Authors:** Takeshi Ara, Yuichi Kodama, Takatomo Fujisawa, Takehide Kosuge, Kyungbum Lee, Jun Mashima, Osamu Ogasawara, Yasuhiro Tanizawa, Tomoya Tanjo, Yasukazu Nakamura, Masanori Arita

**Affiliations:** Bioinformation and DDBJ Center, National Institute of Genetics, Mishima, Shizuoka 411-8540, Japan; Bioinformation and DDBJ Center, National Institute of Genetics, Mishima, Shizuoka 411-8540, Japan; Bioinformation and DDBJ Center, National Institute of Genetics, Mishima, Shizuoka 411-8540, Japan; Bioinformation and DDBJ Center, National Institute of Genetics, Mishima, Shizuoka 411-8540, Japan; Bioinformation and DDBJ Center, National Institute of Genetics, Mishima, Shizuoka 411-8540, Japan; Bioinformation and DDBJ Center, National Institute of Genetics, Mishima, Shizuoka 411-8540, Japan; Bioinformation and DDBJ Center, National Institute of Genetics, Mishima, Shizuoka 411-8540, Japan; Bioinformation and DDBJ Center, National Institute of Genetics, Mishima, Shizuoka 411-8540, Japan; Bioinformation and DDBJ Center, National Institute of Genetics, Mishima, Shizuoka 411-8540, Japan; Bioinformation and DDBJ Center, National Institute of Genetics, Mishima, Shizuoka 411-8540, Japan; Bioinformation and DDBJ Center, National Institute of Genetics, Mishima, Shizuoka 411-8540, Japan

## Abstract

The Bioinformation and DNA Data Bank of Japan Center (https://www.ddbj.nig.ac.jp/) continues to serve as a global core infrastructure for biological information as part of the International Nucleotide Sequence Database Collaboration. In 2024, we reinforced data quality and transparency through mandatory metadata standards, including sampling geolocation and date, aligning with international debates on Digital Sequence Information. Our repositories expanded across multiple omics layers, and our secure environment for analysis of personal genome provides tools and precomputed data on personal genomes archived at the Japanese Genotype-phenotype Archive. International collaboration was advanced through metadata harmonization with the Korea Bioinformation Center and the China National Genomics Data Center, which strengthened regional data resilience and integration. Inside Japan, we began a new collaboration with the Japan Institute of Health Security\, which facilitated the systematic release of pathogen genomes via the Pathogens.jp portal. To support these expanding activities, our high-performance computing infrastructure was renewed with 14 000 CPU cores, 50 PB Lustre storage, and newly deployed GPU nodes. These updates enable both AI-driven analyses and cost-efficient large-scale genome reanalysis.

## Graphical Abstract

**Graphical Abstract:**
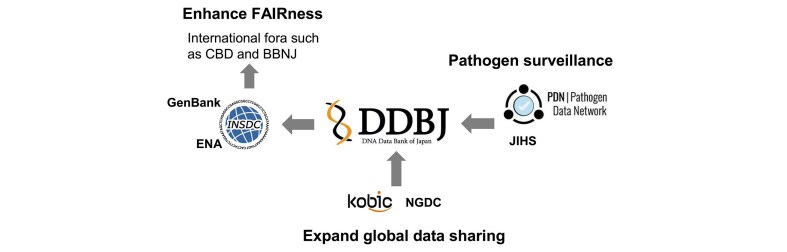

## Introduction

The Bioinformation and DNA Data Bank of Japan (DDBJ) Center (https://www.ddbj.nig.ac.jp) of the National Institute of Genetics is a global-core infrastructure for public biological information. As a founding member of the International Nucleotide Sequence Database Collaboration (INSDC) [1], the DDBJ Center accepts nucleotide sequence data either as raw outputs or assembled/annotated sequences. All data will be assigned unique and persistent accession numbers, and their copies are distributed from all INSDC nodes: the National Center for Biotechnology Information (NCBI) of the National Institutes of Health (NIH) [2] and the European Bioinformatics Institute of European Molecular Biology Laboratory [3].

In addition to INSDC resources, our center also accepts gene expression/transcriptome raw data at GEA (Genomic Expression Archive) [4], metabolome/lipidome raw data at MetaboBank [5], and human genomes and genetic variations at JGA (Japanese Genotype-phenotype Archive) [6, 7]. These repositories are partially exchanging metadata with the other INSDC nodes for data findability. Crosslinkingwith two metadata records is mandatory for large-volume data such as Sequence Read Archive (SRA) for next-generation sequencing (NGS) [2] or MetaboBank for mass spectrometry output: one is the BioProject metadata describing research project and its funding information, and the other is the BioSample metadata describing the bioresource information including the mandatory sampling geolocation and date.

These metadata records help not only organize multi-omics datasets across repositories, enabling interoperability and efficient reuse [8], but are also inline with the ongoing debate on Digital Sequence Information (DSI) in several international fora, including the Convention on Biological Diversity (CBD) for ensuring compliance and transparency. Therefore, we also mandate registration of BioProject and BioSample records for assembled/annotated sequences, but metadata registration is exempted for small data such as single genes, viruses, or organelle genomes of <500 kb. Since May 2023, even such small data submissions to DDBJ through our web interface have mandated reporting of the sampling geolocation and date. The geolocation may be missing for “lab stock,” which indicates a cultured cell line or model organism under long-term laboratory control, or for “endangered species” whose exact location is better to be hidden. For such controlled vocabularies, submitters are advised to check the INSDC website for these controlled terms (https://www.insdc.org/technical-specifications/missing-value-reporting/).

Over the past few years, the DDBJ Center has reinforced its international collaborations. With the Korea Bioinformation Center (KOBIC), metadata standards have been harmonized for the BioProject and BioSample formats, and the metadata registered in the Korea BioData Station are now released in a compatible data structure [9]. We regularly receive their NGS raw reads in SRA format and systematically assign persistent accession numbers. Furthermore, data mirroring was started with the National Genomics Data Center (NGDC) of China, to extend the resilience and accessibility of sequence data from East Asia [10]. These collaborations contribute to globalizing the INSDC framework.

Another focus in 2025 is the data handling of pathogen genomes. DDBJ started official collaboration with the iCROWN project (Infectious Disease Clinical Research NetwOrk With National Repository) of the newly restructured organization of Japan Institute for Health Security (JIHS) and started to publish SARS-CoV-2 and Mpox virus genomes isolated in Japan. To accommodate this change, the original portal site (https://COVID19DataPortal.jp) created by National Institute of Informatics, which supported data publication on SARS-CoV-2 during the pandemic, was renamed and integrated into a broader platform of Pathogens.jp at DDBJ. This new collaborative portal provides a sustainable framework for archiving and analyzing data across a wide range of human and animal pathogens as part of the international Pathogen Data Network (PDN; https://pathogendatanetwork.org/), which is coordinated by the Swiss Institute of Bioinformatics.

To support these expanding activities, the DDBJ Center upgraded its high-performance computing infrastructure in early 2025. Notably, the newly installed GPU nodes include the NVIDIA DGX B200 system, which enables state-of-the-art AI analyses since June. In addition, cost-effective GPU nodes of NVIDIA L40S and PEZY-SC3 processors were introduced for large-scale genome analysis beginning in April. This upgraded infrastructure substantially enhances both computational performance and energy efficiency. We report updates to the databases and the services of the DDBJ Center. All resources are available at https://www.ddbj.nig.ac.jp and the data are accessible at https://ddbj.nig.ac.jp/public/ or ftp://ftp.ddbj.nig.ac.jp. We also describe recent updates to the DDBJ databases, international collaborations, and the upgraded computational infrastructure.

## Data contents and services

### Data contents: unrestricted- and controlled-access databases

The update of data contents in 2024 is summarized in Table 1. Over 100K NGS runs were submitted, the majority of which (>99%) came from Japan, reflecting the strong domestic utilization of the DDBJ services, while international contributions remain modest but steadily increasing. Monthly statistics is also available online with access counts for each service at the bottom of our webpage (https://www.ddbj.nig.ac.jp/statistics/index-e.html). NGS-related contents (SRA and JGA with BioProject and BioSample), except for access-controlled data, are findable through our integrated search interface (https://ddbj.nig.ac.jp/search), and annotated sequences including patented sequences from the Japan Patent Office and the Korean Intellectual Property Office are findable through our traditional search interface (https://ddbj.nig.ac.jp/arsa/ or https://getentry.ddbj.nig.ac.jp/top-e.html). To cite our data, BioProject accession number is useful for referring to the entire dataset, while listing of run or experiment accessions is recommended for spcifying exact information. All datasets are directly accessible at our file server through HTTPS (https://ddbj.nig.ac.jp/public/), where GEA for gene expression and MetaboBank for metabolomics are included. Supercomputer users can access all data directly from their accounts and downloading is unnecessary.

**Table 1.** Data updates in 2024

Submissions in 2024		
Annotated sequences	9603 submissions	87.3% from Japan, 10.8% from Asia, and 1.2% from Africa
NGS reads (SRA)	107 039 runs	99.6% from Japan, 0.3% from Asia, and 0.1% from “Europe and the US”
Genomic expressions (GEA)	207 submissions	205 from Japan, 2 from “Europe and the US”
Metabolome (MetaboBank)	22 studies	All from Japan
Personal genomes (JGA)	107 studies	All from Japan
**Released cumulative total**		
Annotated sequences	5609 M sequences (June 2025 release)	GenBank (80.7%), ENA (15.8%), and DDBJ (3.5%)
NGS reads (DRA)	20.6 PB (Aug 2025)	Including 1.6 PB of FASTQ formats
Gene expressions (GEA)	631 experiments (Aug 2025)	12 829 samples
Metabolome (MetaboBank)	153 studies (Aug 2025)	4145 samples
Personal genomes (JGA)	501 studies	964 235 samples

For data submitters, we encourage registration of BioProject and BioSample records as instructed in the Pathogen Data Object Model [11]. While accurate geolocation and date are important for surveillance, they may also interfere with the privacy of infected subjects. For this reason, our data collaboration with medical institutes including JIHS uses compromised data description such as using a date range rather than a specific day for Mpox virus (Bioproject PRJDB16992), or publishing only assembled, complete genomic data for SARS-CoV-2 without clinical records (DDBJ BS001145–BS016472).

For submitting virus and prokaryote genomes, the DFAST annotation service is gaining popularity (https://dfast.ddbj.nig.ac.jp/). Among annotated bacterial genomes submitted in 2024, 98.5% used DFAST [12]. The service now supports a taxonomy check function based on average nucleotide identity against all NCBI RefSeq genomes (22 171 type genomes) [13] and Genome Taxonomy Database (GTDB) genomes (113 104 representatives of Release 220) [14]. The distance calculation is accelerated using MinHash and sparse chaining, allowing it to be completed within a few minutes [15].

### Personal genomes and their reanalysis

Several sets of human genomes are publicly accessible without restriction: International 1000 Genomes Project (accession number PRJEB31736); the Human Genome Diversity Project (PRJEB6463); the Simons Genome Diversity Project (PRJEB9589, ERP010710); and the Korean Personal Genomics Project (PRJNA284338). For better utilization, we mapped the four datasets using the BWA-MEM algorithm to two new reference genomes, the Build 38 of Human Genome Consortium and the CHM13 of Telomere-to-Telomere Consortium, and called for variants using the HaplotypeCaller in the Best Practices of Genome Analysis Toolkit (GATK4.2) with default options [16]. The resulting CRAM-formatted files and gVCF files, and the joint call results for the four datasets are also available from our repository without access restriction (https://ddbj.nig.ac.jp/public/public-human-genomes/).

For datasets under controlled access, only their summaries are openly accessible at the NBDC Human Database (https://humandbs.dbcls.jp/en/data-use/all-researches). This database began operation in October 2013, and its management body was temporarily transferred to the Database Center for Life Science (DBCLS) at Research Organization of Information and Systems in April 2024 (https://biosciencedbc.jp/en/news/20240401-03.html). This body is planned to integrate with DDBJ in 2026 for more efficient data management. The request for data use is currently handled by the Data Access Committee of DBCLS and actual data access is provided from JGA of DDBJ Center. Since the security requirement for handling personal genomes is strict in Japan (e.g. no use of commercial cloud), DDBJ provides a dedicated hardware district for users for a fee. In this district, each user occupies an independent CPU node to guarantee insulation from other users for security pourposes. Our supercomputer users can access the above-mentioned public data and also access-controlled data (upon request and permission) in their local directory. Imputation service is also available with various reference datasets (https://sc.ddbj.nig.ac.jp/advanced_guides/TogoImputation/imputation_server). Due to our limitation of resources, however, GPU jobs are managed with a dedicated Slurm job scheduler among personal genome users to fully utilize 24 NVIDIA L40S GPUs.

### Integration of login accounts and identifiers

Our databases and repositories require different data access policies. For historical reasons, two incompatible user-identification schemes have been running with heterogeneous specifications and implementations: unrestricted-access systems (such as DDBJ and DFAST) and controlled-access systems (NBDC Human Database and JGA). To resolve this complex authentication and authorization system, we launched a new account service in March 2025 to decouple authentication and authorization, and to modularize the database functions. We standardized the user attributes required for applications and management across all repositories. Through the new system, users can create accounts and manage their attribute information more directly and flexibly. In addition, separation of authentication and authorization will enable the integration of new services more flexibly, such as the introduction of ORCID login.

## Collaborations

### Pathogen surveillance

In the aftermath of the COVID-19 pandemic, Japan recognized the critical importance of building a more efficient and coordinated framework for data sharing. During the pandemic, genomic, epidemiological, and clinical data were often managed in separate silos, resulting in delays in integration and analysis. To address these challenges, national stakeholders initiated discussions on sustainable platforms that could be rapidly mobilized in response to future public health emergencies. In April 2025, the National Center for Global Health and Medicine and the National Institute of Infectious Diseases were integrated to form the JIHS. Along with this institutional reorganization, the national program for infectious diseases previously known as REBIND (REpository of Data and Biospecimen of INfectious Disease) was restructured and relaunched as iCROWN. Under this new framework, genomic data obtained through iCROWN are systematically released via the DDBJ, ensuring open access to high-quality pathogen genome sequences and facilitating international data sharing in accordance with the INSDC standards. This post-pandemic effort aims not only to improve the speed and accuracy of infectious disease surveillance in Japan but also to ensure that the domestic data can be seamlessly incorporated into international data-sharing frameworks. For this purpose, a new portal site pathogens.jp was launched as an Asian node of the international PDN, which is coordinated by the Swiss Institute of Bioinformatics with NIH grant.

### Compliance with the biodiversity framework

In line with the CBD and the ongoing international debate on DSI, INSDC mandates the inclusion of sampling geolocation and sampling date as essential metadata for all submissions. These attributes are considered critical for ensuring transparency of genetic resource utilization. The requirement at DDBJ started on 17 May 2023, but even before this requirement, 71.2% of BioSample records registered at DDBJ within 1 year before the date contained country names (59.5% Japan, 1.5% China, 1.4% Vietnam, 1.2% Pacific Ocean and others; 28.8% no country name in 120 425 records total). After the mandate, 90.9% of BioSample records registered within 1 year after the date contained country names (49.9% Japan, 10.4% US, 6.4% Antarctica, 6.3% China, 2.2% Mexico and others; 9.2% no country name in 166 372 records total). The number of records and country ratio fluctuate year by year, but it is evident that researchers are collaborative to provide provenance information of the genetic resource they utilize. A similar statistics was obtained for the sampling date; >90% of records now contain sampling dates. The qualifier name for the geolocation of assembled/annotated sequences was changed from “country” to “geo_loc_name” in June 2024 to streamline the description of metadata. Marine and island names are updated upon request (https://www.insdc.org/submitting-standards/geo_loc_name-qualifier-vocabulary/).

### Global expansion through data sharing

To further enhance global collaboration, the data mirroring is ongoing with KOBIC in Korea and NGDC in China. As of summer 2025, KOBIC has released domestic information in the INSDC formats, with 400 BioProjects, 10 305 BioSamples, and 20 667 SRA runs mirrored via DDBJ. In parallel, large-scale mirroring of NGDC resources began in 2025, covering 15 880 BioProjects, 722 949 BioSamples, and 17 225 SRA runs so far. These continuing efforts strengthen data findability and interoperability across East Asia, while preparing INSDC for the future global expansion on DSI.

## DDBJ system update

### Supercomputing facility

To accommodate the rapidly growing demand for large-scale data sharing and associated computation (validation and format conversion), particularly for new sequencing data from NGDC, DDBJ completed a major upgrade of its supercomputer system between March–May 2025. In this renewal, a dedicated 50 PB Lustre storage was allocated for DDBJ operations, enabling long-term archiving and high-throughput access to supercomputer users. The new system comprises >14 000 CPU cores, with GPU nodes optimized for bioinformatics and AI applications. Four NVIDIA DGX B200 systems (total 32 B200 GPUs) were introduced on 1 June 2025, as the first deployment at a public institution in Japan, providing high-end performance for AI-driven data analysis for researchers. In addition, cost-effective nodes with NVIDIA L40S and two PEZY-SC3 systems were deployed for large-scale analysis including the reanalysis of open human genomes introduced above.

In Table 2, the performance of L40S comparable to the NVIDIA H100 in GATK-compatible genome analysis pipelines is noteworthy because its cost is roughly one-third, making it highly recommended for population-scale sequencing projects. This is because genome analysis primarily depends on FP32 (32-bit single-precision) performance, unlike AI workloads where GPU memory size, memory bandwidth, and low-precision floating-point formats such as FP8 (8-bit floating point) or FP4 (4-bit floating point) are critical. While high-end GPUs such as NVIDIA H100 and B200 offer superior memory bandwidth and AI-specific acceleration, their advantage is not fully utilized in genome analysis workflows. This suggests that L40S is a practical and sustainable choice for population-scale genomics, allowing public research institutions to allocate resources efficiently while reserving high-end GPUs for AI-oriented applications.

**Table 2.** Benchmarking speed for the GPU nodes using the GATK-compliant software on NA18945 sample from the 1KGP 30x dataset

	NVIDIA V100 (2017)	NVIDIA A100 (2020)	NVIDIA L40S (2022)	NVIDIA H100 (2022)	NVIDIA B200 (2024)	PEZY-SC3
Parabricks run (hh:mm:ss)	3:07:29	2:06:14	1:45:05	1:56:24	N/A	1:02:55
Memory size (GB)	16 or 32	40 or 80	40	80 or 94	192	32
Memory bandwidth (GB/s)	900	2039	864	3352	8000	1200
FP32 (TFlops)	15.7	19.5	91.6	66.9	80	39.32
TF32 tensor core (TFlops)	125	312	366	989	2200	N/A

L40S performs well in spite of its lower cost. PEZY-SC3 is an MIMD processor with 4096 cores (not GPU).

In collaboration with two commercial bodies (PEZY Computing Inc. and Genome Analytics Japan Inc.), we further demonstrated a high-speed human genome analysis on the ZettaVEGA system customized for the unique PEZY-SC3 architecture, which achieved a 2.8-fold acceleration (33 min on average) over the computing node with four V100 GPUs (90 min), and even faster than the node with eight H100 GPUs (37 min) while delivering 99.9995% concordance with the GATK4.1 reference results (data for GRGh38 and CHM13). The whitepaper of this system is available from its company website (https://www.pezy.co.jp/wp-content/uploads/2025/09/zettavega_whitepaper202509.pdf).

## Future directions

In the coming years, the DDBJ Center needs to strengthen the interoperability of its databases, expand collaborations for pathogen surveillance, and provide AI-driven analytical services on its upgraded HPC infrastructure. Regional collaboration with neighboring countries beyond East Asia is especially important to globalize data sharing and to improve transparency of utilization of genetic resources. We also need to be aware of both benefits and risks of using AI tools in our curation and data management activities. These efforts will ensure that the DDBJ continues to function as a trusted node of the INSDC.

## Data Availability

All services are available at https://www.ddbj.nig.ac.jp/ and the data are accessible at https://ddbj.nig.ac.jp/public/ or ftp://ftp.ddbj.nig.ac.jp/.
